# μ-Squarato-κ^2^ *O* ^1^:*O* ^2^-bis­{[2-(2-amino­ethyl)pyridine-κ^2^ *N*,*N*′]aqua­nickel(II)} squarate 0.25-hydrate

**DOI:** 10.1107/S1600536808030808

**Published:** 2008-09-27

**Authors:** Yunus Bekdemir, Ibrahim Uçar, Orhan Büyükgüngör

**Affiliations:** a Ondokuz Mayıs University, Art and Science Faculty, Department of Chemistry, 55139 Samsun, Turkey; b Ondokuz Mayıs University, Art and Science Faculty, Department of Physics, 55139 Samsun, Turkey

## Abstract

The asymmetric unit of title compound, [Ni_2_(C_4_O_4_)(C_7_H_10_N_2_)_4_(H_2_O)_2_]C_4_O_4_·0.25H_2_O, contains one-half of a squarate ligand, one-half of an uncoordinated squarate dianion, two 2-(2-amino­ethyl)pyridine ligands and one aqua ligand, all coordinated to an Ni^II^ ion. The compound also contains 0.25 solvent water mol­ecules. The Ni^II^ ion has distorted octa­hedral geometry. The squarate ligand adopts a μ-1,2 coordination mode, the intra­dimer Ni^II^⋯Ni^II^ separation being 7.1442 (7) Å, while the other squarate unit acts as a counter-anion. The crystal structure is stabilized by inter­molecular O—H⋯O and N—H⋯O hydrogen-bond inter­actions, forming a three-dimensional network.

## Related literature

For general background, see: Bernardinelli *et al.* (1989 ▶); Bulut *et al.* (2004 ▶); Castro *et al.* (1995 ▶, 1997 ▶); Crispini *et al.* (2000 ▶); Kirchmaier *et al.* (2003 ▶); Lee *et al.* (1996 ▶); Milet *et al.* (2003 ▶); Solans *et al.* (1990 ▶); Spek (2003 ▶); Trombe *et al.* (2002 ▶); Uçar (2008 ▶); Uçar *et al.* (2006 ▶, 2007 ▶); Yang *et al.* (2003 ▶).
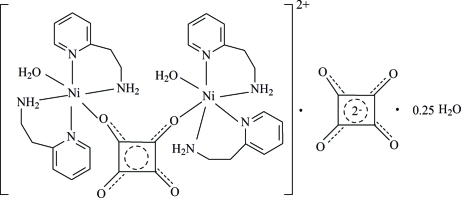


## Experimental

### 

#### Crystal data

[Ni_2_(C_4_O_4_)(C_7_H_10_N_2_)_4_(H_2_O)_2_]C_4_O_4_·0.25H_2_O
*M* *_r_* = 870.16
Monoclinic, 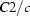

*a* = 28.037 (3) Å
*b* = 8.0409 (5) Å
*c* = 17.7752 (16) Å
β = 103.572 (7)°
*V* = 3895.3 (5) Å^3^
*Z* = 4
Mo *K*α radiation
μ = 1.03 mm^−1^
*T* = 297 (2) K
0.3 × 0.2 × 0.1 mm

#### Data collection

Stoe IPDSII diffractometer
Absorption correction: integration (*X-RED32*; Stoe & Cie, 2002 ▶) *T*_min_ = 0.49, *T*_max_ = 0.81
12243 measured reflections
3809 independent reflections
3148 reflections with *I* > 2σ(*I*)
*R*_int_ = 0.042

#### Refinement

*R*[*F* ^2^ > 2σ(*F* ^2^)] = 0.029
*wR*(*F* ^2^) = 0.073
*S* = 1.03
3809 reflections
282 parameters
H atoms treated by a mixture of independent and constrained refinement
Δρ_max_ = 0.28 e Å^−3^
Δρ_min_ = −0.26 e Å^−3^

### 

Data collection: *X-AREA* (Stoe & Cie, 2002 ▶); cell refinement: *X-AREA*; data reduction: *X-RED32* (Stoe & Cie, 2002 ▶); program(s) used to solve structure: *SHELXS97* (Sheldrick, 2008 ▶); program(s) used to refine structure: *SHELXL97* (Sheldrick, 2008 ▶); molecular graphics: *ORTEPIII* (Burnett & Johnson, 1996 ▶); software used to prepare material for publication: *WinGX* (Farrugia, 1999 ▶).

## Supplementary Material

Crystal structure: contains datablocks I, global. DOI: 10.1107/S1600536808030808/pk2116sup1.cif

Structure factors: contains datablocks I. DOI: 10.1107/S1600536808030808/pk2116Isup2.hkl

Additional supplementary materials:  crystallographic information; 3D view; checkCIF report

## Figures and Tables

**Table 1:** Hydrogen-bond geometry (Å, °)

*D*—H⋯*A*	*D*—H	H⋯*A*	*D*⋯*A*	*D*—H⋯*A*
O5—H5*A*⋯O4^i^	0.81 (3)	1.93 (3)	2.716 (2)	163 (3)
N2—H2*A*⋯O3^i^	0.90 (2)	2.09 (2)	2.935 (2)	157 (2)
O5—H5*B*⋯O2	0.81 (3)	1.87 (3)	2.675 (2)	172 (3)
N4—H4*B*⋯O2^ii^	0.88 (2)	2.52 (2)	3.078 (2)	122.6 (18)
N4—H4*B*⋯O6	0.88 (2)	2.52 (3)	3.350 (2)	158 (2)
N4—H4*A*⋯O4^iii^	0.86 (2)	2.27 (2)	3.091 (2)	161 (2)
N2—H2*B*⋯O3^iv^	0.83 (2)	2.14 (3)	2.963 (2)	169 (2)

Symmetry codes: (i) 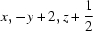; (ii) 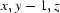; (iii) 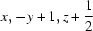; (iv) 
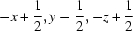
.

**Fig. 1.**
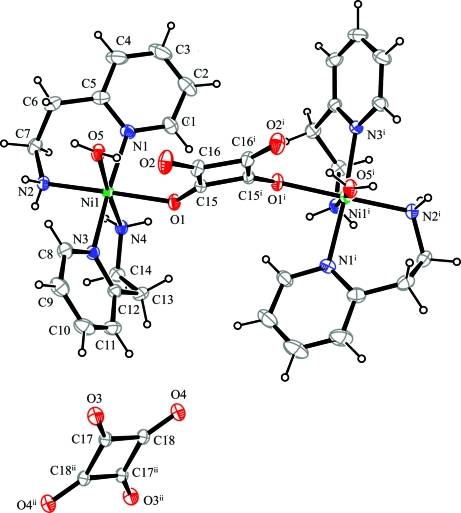
: ORTEPIII (Burnett & Johnson, 1996) plot of the [Ni2(aepy)4(sq)(H2O)2].sq 0.25H2O. Non-H atoms are drawn with displacement ellipsoids at the 30% probability level and H atoms are shown as small spheres of arbitrary radii. [Symmetry codes: (i) -x, y, -z + 1/2; (ii) -x + 1/2, -y + 1.5, -z]

**Fig. 2.**
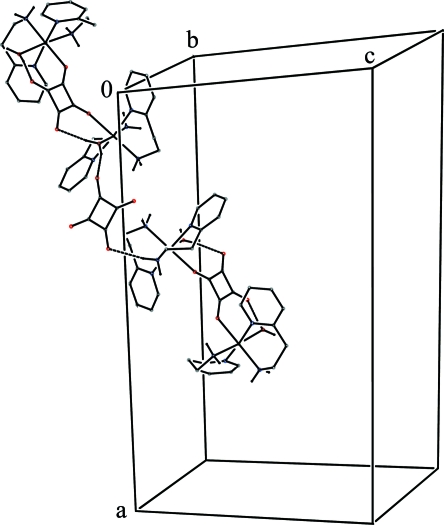
: Showing of intermolecular hydrogen bonding interactions (dashed lines) in the unitcell.

## supplementary crystallographic information

### Comment

Squarate acts as a bridge between two or more metal atoms in mono- or polydentate coordination modes when acting as a ligand towards first row transition metal ions [Trombe *et al.*, 2002; Milet *et al.*, 2003]. It coordinates to Fe(II), Fe(III), Ni^II^ and Cu(II) complexes in a µ-1,3 fashion, giving binuclear [Bernardinelli *et al.*, 1989; Lee *et al.*, 1996] and chain structures [Solans *et al.*, 1990; Yang *et al.*,2003], whereas the µ-1,2 coordination mode has been reported for binuclear and chain complexes of Cu(II) and Pd(II) [Castro *et al.*, 1997; Crispini *et al.*, 2000]. It is also observed that the squarate anion, with Cu(II) and Ni^II^, acts as a tetramonodentate ligand and forms polynuclear compounds [Castro *et al.*, 1995].

In our ongoing research on squaric acid, we have synthesized some mixed–ligand metal(II) complexes of squaric acid, and their structures have been reported [Uçar *et al.*, 2006; Uçar *et al.* 2007;]. In these compounds, squaric acid behaves as a monodentate ligand [Bulut *et al.*, 2004] or acts as both a monodentate and bidentate ligand, or has a µ-1,3 coordination, while in this study, it has µ-1,2 bis(monodentate) coordination between the metal ions.

The title compound consists of an apparently centrosymmetric binuclear [Ni_2_(aepy)_4_(H_2_O)_2_(C_4_O_4_)]^2+^ [aepy: 2(2-aminoethyl)pyridine] complex cation, one squarate counter anion (C_4_O_4_)^2-^and 0.25 water molecule. In the crystal structure one of the squarates adopts a bridging position between the metal atoms, coordinating *via* two of its O atoms in a µ-1,2 fashion, forming a dimeric metal unit, while the other squarate acts as a counter anion (Fig.1). The geometry about Ni^II^ ion centre is a slightly distorted octahedron, the six coordination sites being occupied by four N atoms from two chelating aepy ligands and two O atoms from squarate and aqua ligands. The observed Ni1–N, Ni1–O bond distances and N–Ni1–N, N–Ni1–O and O–Ni1–O bond angles are generally consistent with those observed in related Ni^II^ squarate complexes [Uçar, 2008; Kirchmaier *et al.*, 2003].

The crystal packing is formed by intermolecular hydrogen bonding interactions (Fig. 2). The aqua ligands and amine hydrogen atoms link the complex cation to counter the squarate anion through hydrogen bonding interactions. The intradimer Ni1(II)···Ni1 (-*x*, *y*, -*z* + 1/2) distance is 7.1442 (7) Å.

### Experimental

Squaric acid (0.57 g, 5 mmol), dissolved in 25 ml water was neutralized with NaOH (0.40 g, 10 mmol) and was added to a hot solution of the NiCl_2_.6H_2_O (1.19 g, 5 mmol) dissolved in 100 ml water. The mixture was refluxed at 353 K for 12 h and then cooled to room temperature.  The blue crystals that formed were filtered and washed with water and alcohol and dried in vacuum. A solution of 2(2-aminoethyl)pyridine) (0.25 g, 2 mmol) in ethanol (50 ml) was added dropwise with stirring to a suspension of the NiSq.2H_2_O (0.207 g, 1 mmol) in water (100 ml). A few days later, well formed blue crystals were selected for X-ray studies.

### Refinement

H atoms attached to C atoms were placed at calculated positions (C—H=0.93 and 0.97 Å) and were allovwed to ride on the parent atom [*U*_iso_(H)=1.2_eq_(C)]. The remaining H atoms were located in a difference map. At this stage, the maximum difference density of 1.31 e Å^-3^ indicated the presence of a possible atom site. A check of the solvent-accessible volume using *PLATON* (Spek, 2003) showed a total potential volume of 33.0 Å^3^. Attempts to refine this peak as a water O atom (O6) resulted in a partial occupancy of 0.12. For the final cycle of refinement the occupancy of O6 was fixed at 0.125. H atoms attached to O6 were not located.

### Crystal data

**Table:**

[Ni_2_(C_4_O_4_)(C_7_H_10_N_2_)_4_(H_2_O)_2_]C_4_O_4_·0.25H_2_O	*F*(000) = 1816
*M**_r_* = 870.16	*D*_x_ = 1.484 Mg m^−^^3^
Monoclinic, *C*2/*c*	Mo *K*α radiation, λ = 0.71069 Å
Hall symbol: -C 2yc	Cell parameters from 24222 reflections
*a* = 28.037 (3) Å	θ = 1.7–28.0°
*b* = 8.0409 (5) Å	µ = 1.03 mm^−^^1^
*c* = 17.7752 (16) Å	*T* = 297 K
β = 103.572 (7)°	Prism, blue
*V* = 3895.3 (5) Å^3^	0.3 × 0.2 × 0.1 mm
*Z* = 4	

### Data collection

**Table:**

Stoe IPDS-II diffractometer	3809 independent reflections
Radiation source: fine-focus sealed tube	3148 reflections with *I* > 2σ(*I*)
graphite	*R*_int_ = 0.042
Detector resolution: 6.67 pixels mm^-1^	θ_max_ = 26.0°, θ_min_ = 2.4°
ω scans	*h* = −34→34
Absorption correction: integration (X-RED32; Stoe & Cie, 2002)	*k* = −9→9
*T*_min_ = 0.49, *T*_max_ = 0.81	*l* = −21→21
12243 measured reflections	

### Refinement

**Table:**

Refinement on *F*^2^	Primary atom site location: structure-invariant direct methods
Least-squares matrix: full	Secondary atom site location: difference Fourier map
*R*[*F*^2^ > 2σ(*F*^2^)] = 0.029	Hydrogen site location: inferred from neighbouring sites
*wR*(*F*^2^) = 0.073	H atoms treated by a mixture of independent and constrained refinement
*S* = 1.03	*w* = 1/[σ^2^(*F*_o_^2^) + (0.0385*P*)^2^ + 0.9229*P*] where *P* = (*F*_o_^2^ + 2*F*_c_^2^)/3
3809 reflections	(Δ/σ)_max_ = 0.001
282 parameters	Δρ_max_ = 0.29 e Å^−^^3^
0 restraints	Δρ_min_ = −0.26 e Å^−^^3^

### Special details

**Table:**

Geometry. All e.s.d.'s (except the e.s.d. in the dihedral angle between two l.s. planes) are estimated using the full covariance matrix. The cell e.s.d.'s are taken into account individually in the estimation of e.s.d.'s in distances, angles and torsion angles; correlations between e.s.d.'s in cell parameters are only used when they are defined by crystal symmetry. An approximate (isotropic) treatment of cell e.s.d.'s is used for estimating e.s.d.'s involving l.s. planes.
Refinement. Refinement of *F*^2^ against ALL reflections. The weighted *R*-factor *wR* and goodness of fit *S* are based on *F*^2^, conventional *R*-factors *R* are based on *F*, with *F* set to zero for negative *F*^2^. The threshold expression of *F*^2^ > σ(*F*^2^) is used only for calculating *R*-factors(gt) *etc*. and is not relevant to the choice of reflections for refinement. *R*-factors based on *F*^2^ are statistically about twice as large as those based on *F*, and *R*- factors based on ALL data will be even larger.

### Fractional atomic coordinates and isotropic or equivalent isotropic displacement parameters (Å^2^)

**Table:**

	*x*	*y*	*z*	*U*_iso_*/*U*_eq_	Occ. (<1)
C1	0.01456 (9)	0.6924 (3)	0.42515 (16)	0.0615 (6)	
H1	0.0078	0.6622	0.3732	0.074*	
C2	−0.02378 (11)	0.7007 (4)	0.4607 (2)	0.0880 (9)	
H2	−0.0557	0.6790	0.4331	0.106*	
C3	−0.01433 (14)	0.7411 (5)	0.5371 (3)	0.1052 (12)	
H3	−0.0396	0.7486	0.5627	0.126*	
C4	0.03335 (14)	0.7707 (4)	0.5755 (2)	0.0885 (10)	
H4	0.0406	0.7952	0.6281	0.106*	
C5	0.07099 (10)	0.7643 (3)	0.53672 (15)	0.0568 (6)	
C6	0.12298 (10)	0.7973 (3)	0.57849 (13)	0.0590 (6)	
H6A	0.1253	0.7886	0.6337	0.071*	
H6B	0.1311	0.9109	0.5679	0.071*	
C7	0.16091 (9)	0.6827 (3)	0.55784 (13)	0.0535 (5)	
H7A	0.1905	0.6846	0.5990	0.064*	
H7B	0.1485	0.5697	0.5529	0.064*	
C8	0.18856 (8)	0.9557 (3)	0.32778 (14)	0.0528 (5)	
H8	0.1919	1.0099	0.3749	0.063*	
C9	0.21215 (9)	1.0213 (3)	0.27497 (17)	0.0667 (7)	
H9	0.2311	1.1169	0.2865	0.080*	
C10	0.20735 (10)	0.9436 (3)	0.20518 (17)	0.0685 (7)	
H10	0.2223	0.9864	0.1679	0.082*	
C11	0.17984 (9)	0.8010 (3)	0.19165 (14)	0.0586 (6)	
H11	0.1759	0.7462	0.1445	0.070*	
C12	0.15786 (8)	0.7380 (2)	0.24758 (12)	0.0445 (4)	
C13	0.13096 (9)	0.5757 (3)	0.23578 (13)	0.0539 (5)	
H13A	0.1395	0.5175	0.1929	0.065*	
H13B	0.0960	0.5978	0.2216	0.065*	
C14	0.14211 (8)	0.4629 (2)	0.30683 (13)	0.0498 (5)	
H14A	0.1344	0.3488	0.2908	0.060*	
H14B	0.1768	0.4686	0.3313	0.060*	
C15	0.02527 (7)	0.9053 (2)	0.27139 (11)	0.0361 (4)	
C16	0.02560 (8)	1.0864 (2)	0.27195 (12)	0.0452 (5)	
C17	0.24949 (7)	0.8704 (2)	0.01973 (11)	0.0375 (4)	
C18	0.21239 (7)	0.7408 (2)	−0.00476 (11)	0.0375 (4)	
N1	0.06135 (7)	0.7253 (2)	0.46111 (11)	0.0472 (4)	
N2	0.17263 (6)	0.7340 (2)	0.48476 (10)	0.0404 (4)	
N3	0.16111 (6)	0.81826 (19)	0.31494 (9)	0.0396 (4)	
N4	0.11369 (7)	0.51155 (19)	0.36278 (11)	0.0407 (4)	
O1	0.05537 (5)	0.79065 (16)	0.29572 (8)	0.0449 (3)	
O2	0.05610 (6)	1.19590 (18)	0.29910 (11)	0.0663 (5)	
O3	0.24837 (5)	1.01589 (15)	0.04408 (9)	0.0487 (4)	
O4	0.16716 (5)	0.73117 (15)	−0.01038 (9)	0.0470 (3)	
O5	0.10943 (6)	1.01843 (17)	0.41638 (11)	0.0479 (4)	
O6	0.0000	0.4863 (13)	0.2500	0.121 (6)	0.25
Ni1	0.114036 (8)	0.76294 (2)	0.391260 (13)	0.03314 (8)	
H5A	0.1284 (10)	1.079 (3)	0.4454 (16)	0.062 (8)*	
H5B	0.0958 (10)	1.073 (3)	0.3792 (16)	0.062 (8)*	
H2A	0.1897 (8)	0.829 (3)	0.4946 (13)	0.050 (6)*	
H4B	0.0829 (9)	0.485 (3)	0.3435 (14)	0.052 (6)*	
H4A	0.1238 (8)	0.454 (3)	0.4042 (13)	0.043 (6)*	
H2B	0.1917 (9)	0.666 (3)	0.4725 (14)	0.051 (6)*	

### Atomic displacement parameters (Å^2^)

**Table:**

	*U*^11^	*U*^22^	*U*^33^	*U*^12^	*U*^13^	*U*^23^
C1	0.0446 (13)	0.0681 (13)	0.0736 (17)	−0.0058 (11)	0.0174 (12)	0.0051 (12)
C2	0.0501 (17)	0.107 (2)	0.115 (3)	−0.0029 (15)	0.0356 (18)	0.005 (2)
C3	0.077 (2)	0.125 (3)	0.135 (3)	0.0032 (19)	0.069 (2)	−0.012 (2)
C4	0.096 (2)	0.102 (2)	0.085 (2)	0.0014 (18)	0.0552 (19)	−0.0172 (17)
C5	0.0688 (15)	0.0494 (11)	0.0581 (14)	0.0021 (10)	0.0271 (12)	−0.0041 (10)
C6	0.0784 (18)	0.0595 (12)	0.0405 (12)	−0.0028 (12)	0.0168 (12)	−0.0056 (10)
C7	0.0616 (15)	0.0523 (11)	0.0414 (12)	0.0008 (10)	0.0017 (10)	0.0058 (9)
C8	0.0499 (12)	0.0523 (11)	0.0573 (14)	−0.0113 (10)	0.0150 (10)	−0.0018 (10)
C9	0.0566 (14)	0.0622 (13)	0.086 (2)	−0.0107 (11)	0.0271 (14)	0.0122 (13)
C10	0.0680 (16)	0.0727 (15)	0.0763 (18)	0.0119 (13)	0.0403 (14)	0.0257 (14)
C11	0.0643 (15)	0.0691 (14)	0.0469 (13)	0.0177 (12)	0.0221 (11)	0.0091 (11)
C12	0.0422 (11)	0.0507 (10)	0.0412 (10)	0.0092 (8)	0.0109 (9)	0.0028 (9)
C13	0.0606 (14)	0.0580 (12)	0.0442 (12)	−0.0021 (10)	0.0148 (10)	−0.0159 (10)
C14	0.0523 (12)	0.0358 (9)	0.0623 (14)	0.0000 (8)	0.0152 (10)	−0.0090 (9)
C15	0.0335 (10)	0.0379 (9)	0.0354 (10)	−0.0017 (7)	0.0048 (8)	−0.0008 (7)
C16	0.0482 (12)	0.0399 (9)	0.0420 (11)	−0.0030 (8)	−0.0004 (9)	0.0023 (8)
C17	0.0379 (10)	0.0355 (8)	0.0371 (10)	0.0011 (7)	0.0046 (9)	0.0034 (7)
C18	0.0391 (10)	0.0341 (8)	0.0368 (9)	0.0013 (7)	0.0036 (8)	0.0045 (7)
N1	0.0442 (10)	0.0479 (9)	0.0529 (10)	−0.0028 (7)	0.0181 (8)	−0.0007 (8)
N2	0.0373 (9)	0.0374 (8)	0.0429 (9)	0.0007 (7)	0.0023 (7)	−0.0019 (7)
N3	0.0379 (9)	0.0415 (8)	0.0390 (9)	−0.0037 (6)	0.0085 (7)	0.0004 (7)
N4	0.0386 (10)	0.0356 (8)	0.0447 (10)	−0.0031 (7)	0.0033 (8)	−0.0010 (7)
O1	0.0379 (7)	0.0416 (7)	0.0484 (8)	0.0058 (6)	−0.0039 (6)	−0.0029 (6)
O2	0.0651 (11)	0.0435 (7)	0.0744 (12)	−0.0173 (7)	−0.0155 (9)	0.0054 (7)
O3	0.0471 (8)	0.0340 (6)	0.0627 (10)	0.0011 (6)	0.0083 (7)	−0.0049 (6)
O4	0.0369 (7)	0.0426 (7)	0.0590 (9)	0.0006 (6)	0.0061 (7)	0.0018 (6)
O5	0.0455 (8)	0.0366 (7)	0.0543 (9)	0.0007 (6)	−0.0032 (7)	−0.0056 (7)
O6	0.081 (8)	0.056 (6)	0.196 (16)	0.000	−0.027 (9)	0.000
Ni1	0.03027 (13)	0.03237 (12)	0.03530 (13)	−0.00067 (9)	0.00469 (9)	−0.00121 (9)

### Geometric parameters (Å, °)

**Table:**

C1—N1	1.344 (3)	C13—C14	1.527 (3)
C1—C2	1.371 (4)	C13—H13A	0.9700
C1—H1	0.9300	C13—H13B	0.9700
C2—C3	1.361 (5)	C14—N4	1.466 (3)
C2—H2	0.9300	C14—H14A	0.9700
C3—C4	1.371 (5)	C14—H14B	0.9700
C3—H3	0.9300	C15—O1	1.256 (2)
C4—C5	1.391 (4)	C15—C15^i^	1.442 (4)
C4—H4	0.9300	C15—C16	1.456 (3)
C5—N1	1.344 (3)	C16—O2	1.243 (2)
C5—C6	1.496 (4)	C16—C16^i^	1.464 (4)
C6—C7	1.516 (3)	C17—O3	1.250 (2)
C6—H6A	0.9700	C17—C18	1.465 (2)
C6—H6B	0.9700	C17—C18^ii^	1.465 (3)
C7—N2	1.471 (3)	C18—O4	1.251 (2)
C7—H7A	0.9700	C18—C17^ii^	1.465 (3)
C7—H7B	0.9700	N1—Ni1	2.1624 (17)
C8—N3	1.336 (3)	N2—Ni1	2.0576 (17)
C8—C9	1.374 (3)	N2—H2A	0.90 (2)
C8—H8	0.9300	N2—H2B	0.83 (2)
C9—C10	1.367 (4)	N3—Ni1	2.1495 (16)
C9—H9	0.9300	N4—Ni1	2.0833 (15)
C10—C11	1.372 (4)	N4—H4B	0.88 (2)
C10—H10	0.9300	N4—H4A	0.86 (2)
C11—C12	1.383 (3)	O1—Ni1	2.0803 (13)
C11—H11	0.9300	O5—Ni1	2.1126 (14)
C12—N3	1.344 (3)	O5—H5A	0.81 (3)
C12—C13	1.497 (3)	O5—H5B	0.81 (3)
			
N1—C1—C2	123.7 (3)	C13—C14—H14B	109.4
N1—C1—H1	118.2	H14A—C14—H14B	108.0
C2—C1—H1	118.2	O1—C15—C15^i^	132.78 (10)
C3—C2—C1	118.8 (3)	O1—C15—C16	136.79 (18)
C3—C2—H2	120.6	C15^i^—C15—C16	90.42 (11)
C1—C2—H2	120.6	O2—C16—C15	135.5 (2)
C2—C3—C4	118.5 (3)	O2—C16—C16^i^	134.90 (12)
C2—C3—H3	120.8	C15—C16—C16^i^	89.58 (11)
C4—C3—H3	120.8	O3—C17—C18	134.03 (18)
C3—C4—C5	120.7 (3)	O3—C17—C18^ii^	135.44 (18)
C3—C4—H4	119.6	C18—C17—C18^ii^	90.52 (15)
C5—C4—H4	119.6	O4—C18—C17^ii^	135.81 (17)
N1—C5—C4	120.4 (3)	O4—C18—C17	134.71 (17)
N1—C5—C6	118.8 (2)	C17^ii^—C18—C17	89.48 (15)
C4—C5—C6	120.8 (3)	C1—N1—C5	117.8 (2)
C5—C6—C7	115.41 (19)	C1—N1—Ni1	118.46 (16)
C5—C6—H6A	108.4	C5—N1—Ni1	122.50 (15)
C7—C6—H6A	108.4	C7—N2—Ni1	116.32 (14)
C5—C6—H6B	108.4	C7—N2—H2A	106.6 (15)
C7—C6—H6B	108.4	Ni1—N2—H2A	110.4 (14)
H6A—C6—H6B	107.5	C7—N2—H2B	109.6 (17)
N2—C7—C6	110.98 (18)	Ni1—N2—H2B	107.7 (17)
N2—C7—H7A	109.4	H2A—N2—H2B	106 (2)
C6—C7—H7A	109.4	C8—N3—C12	117.74 (18)
N2—C7—H7B	109.4	C8—N3—Ni1	118.55 (14)
C6—C7—H7B	109.4	C12—N3—Ni1	122.65 (13)
H7A—C7—H7B	108.0	C14—N4—Ni1	116.85 (12)
N3—C8—C9	123.5 (2)	C14—N4—H4B	108.3 (15)
N3—C8—H8	118.3	Ni1—N4—H4B	106.0 (14)
C9—C8—H8	118.3	C14—N4—H4A	108.5 (14)
C10—C9—C8	119.0 (2)	Ni1—N4—H4A	109.3 (14)
C10—C9—H9	120.5	H4B—N4—H4A	108 (2)
C8—C9—H9	120.5	C15—O1—Ni1	134.29 (12)
C9—C10—C11	118.2 (2)	Ni1—O5—H5A	131.0 (18)
C9—C10—H10	120.9	Ni1—O5—H5B	113.3 (18)
C11—C10—H10	120.9	H5A—O5—H5B	108 (3)
C10—C11—C12	120.5 (2)	N2—Ni1—O1	179.19 (7)
C10—C11—H11	119.8	N2—Ni1—N4	92.52 (7)
C12—C11—H11	119.8	O1—Ni1—N4	87.01 (6)
N3—C12—C11	121.1 (2)	N2—Ni1—O5	90.91 (7)
N3—C12—C13	118.03 (18)	O1—Ni1—O5	89.58 (6)
C11—C12—C13	120.9 (2)	N4—Ni1—O5	176.22 (7)
C12—C13—C14	113.80 (18)	N2—Ni1—N3	92.36 (7)
C12—C13—H13A	108.8	O1—Ni1—N3	86.99 (6)
C14—C13—H13A	108.8	N4—Ni1—N3	90.91 (7)
C12—C13—H13B	108.8	O5—Ni1—N3	90.53 (6)
C14—C13—H13B	108.8	N2—Ni1—N1	92.52 (7)
H13A—C13—H13B	107.7	O1—Ni1—N1	88.16 (7)
N4—C14—C13	111.35 (17)	N4—Ni1—N1	92.23 (7)
N4—C14—H14A	109.4	O5—Ni1—N1	86.03 (6)
C13—C14—H14A	109.4	N3—Ni1—N1	174.08 (6)
N4—C14—H14B	109.4		
			
N1—C1—C2—C3	−1.4 (5)	C13—C12—N3—C8	174.5 (2)
C1—C2—C3—C4	−0.5 (5)	C11—C12—N3—Ni1	164.32 (16)
C2—C3—C4—C5	1.9 (5)	C13—C12—N3—Ni1	−17.4 (3)
C3—C4—C5—N1	−1.5 (4)	C13—C14—N4—Ni1	−48.4 (2)
C3—C4—C5—C6	179.7 (3)	C15^i^—C15—O1—Ni1	−148.2 (2)
N1—C5—C6—C7	−40.5 (3)	C16—C15—O1—Ni1	33.2 (4)
C4—C5—C6—C7	138.4 (2)	C7—N2—Ni1—N4	−85.75 (15)
C5—C6—C7—N2	79.8 (3)	C7—N2—Ni1—O5	92.66 (15)
N3—C8—C9—C10	0.4 (4)	C7—N2—Ni1—N3	−176.77 (14)
C8—C9—C10—C11	−1.3 (4)	C7—N2—Ni1—N1	6.59 (15)
C9—C10—C11—C12	−0.3 (4)	C15—O1—Ni1—N4	168.45 (19)
C10—C11—C12—N3	2.9 (3)	C15—O1—Ni1—O5	−9.92 (19)
C10—C11—C12—C13	−175.3 (2)	C15—O1—Ni1—N3	−100.47 (19)
N3—C12—C13—C14	−42.1 (3)	C15—O1—Ni1—N1	76.12 (19)
C11—C12—C13—C14	136.1 (2)	C14—N4—Ni1—N2	−92.56 (16)
C12—C13—C14—N4	80.6 (2)	C14—N4—Ni1—O1	86.78 (16)
O1—C15—C16—O2	−2.4 (5)	C14—N4—Ni1—N3	−0.16 (16)
C15^i^—C15—C16—O2	178.7 (3)	C14—N4—Ni1—N1	174.82 (16)
O1—C15—C16—C16^i^	178.5 (2)	C8—N3—Ni1—N2	−64.13 (16)
C15^i^—C15—C16—C16^i^	−0.4 (2)	C12—N3—Ni1—N2	127.95 (16)
O3—C17—C18—O4	0.7 (4)	C8—N3—Ni1—O1	116.36 (16)
C18^ii^—C17—C18—O4	−179.9 (3)	C12—N3—Ni1—O1	−51.57 (15)
O3—C17—C18—C17^ii^	−179.4 (3)	C8—N3—Ni1—N4	−156.69 (16)
C18^ii^—C17—C18—C17^ii^	0.0	C12—N3—Ni1—N4	35.39 (16)
C2—C1—N1—C5	1.8 (4)	C8—N3—Ni1—O5	26.81 (16)
C2—C1—N1—Ni1	−165.8 (2)	C12—N3—Ni1—O5	−141.12 (16)
C4—C5—N1—C1	−0.4 (3)	C1—N1—Ni1—N2	−164.45 (17)
C6—C5—N1—C1	178.5 (2)	C5—N1—Ni1—N2	28.56 (17)
C4—C5—N1—Ni1	166.70 (19)	C1—N1—Ni1—O1	15.11 (16)
C6—C5—N1—Ni1	−14.4 (3)	C5—N1—Ni1—O1	−151.88 (17)
C6—C7—N2—Ni1	−52.9 (2)	C1—N1—Ni1—N4	−71.83 (17)
C9—C8—N3—C12	2.1 (3)	C5—N1—Ni1—N4	121.19 (17)
C9—C8—N3—Ni1	−166.43 (19)	C1—N1—Ni1—O5	104.81 (17)
C11—C12—N3—C8	−3.7 (3)	C5—N1—Ni1—O5	−62.18 (17)

Symmetry codes: (i) −*x*, *y*, −*z*+1/2; (ii) −*x*+1/2, −*y*+3/2, −*z*.

### Hydrogen-bond geometry (Å, °)

**Table:**

*D*—H···*A*	*D*—H	H···*A*	*D*···*A*	*D*—H···*A*
O5—H5A···O4^iii^	0.81 (3)	1.93 (3)	2.716 (2)	163 (3)
N2—H2A···O3^iii^	0.90 (2)	2.09 (2)	2.935 (2)	157 (2)
O5—H5B···O2	0.81 (3)	1.87 (3)	2.675 (2)	172 (3)
N4—H4B···O2^iv^	0.88 (2)	2.52 (2)	3.078 (2)	122.6 (18)
N4—H4B···O6	0.88 (2)	2.52 (3)	3.350 (2)	158 (2)
N4—H4A···O4^v^	0.86 (2)	2.27 (2)	3.091 (2)	161 (2)
N2—H2B···O3^vi^	0.83 (2)	2.14 (3)	2.963 (2)	169 (2)

Symmetry codes: (iii) *x*, −*y*+2, *z*+1/2; (iv) *x*, *y*−1, *z*; (v) *x*, −*y*+1, *z*+1/2; (vi) −*x*+1/2, *y*−1/2, −*z*+1/2.
